# Developing the i-MoMCARE application in Cambodia: an iterative co-design approach using rapid prototyping

**DOI:** 10.1136/bmjdh-2026-000074

**Published:** 2026-06-10

**Authors:** Hendra Goh, Chhavarath Dary, Mengieng Ung, Dyna Khuon, Sreymom Oy, Sveng Chea Ath Chhay, Chhorlika Khim, Yan Fang Lee, Rattana Kim, Vonthanak Saphonn, Siyan Yi

**Affiliations:** 1 Saw Swee Hock School of Public Health, National University of Singapore and National University Health System, Singapore; 2 University of Health Sciences, Phnom Penh, Cambodia; 3 SingHealth Duke-NUS Global Health Institute, Singapore; 4 National Maternal and Child Health Center, Phnom Penh, Cambodia

**Keywords:** Global Health, Primary Health Care

## Abstract

**Objective:**

To develop a mobile health (mHealth) application (i-MoMCARE app) to support village health support groups (VHSGs) and health centre staff in delivering maternal and child health (MCH) services in rural Cambodia.

**Methods and analysis:**

We adopted a co-design strategy to guide the development of the i-MoMCARE app. In phase 1, key stakeholders identified and proposed app features based on research-informed end-user needs. Phase 2 involved prioritising high-impact features for app development. In phase 3, VHSGs and health centre staff participated in usability testing of the i-MoMCARE app. Interview data collected through rapid qualitative interviews were thematically analysed.

**Results:**

Sixteen stakeholders participated in phases 1 and 2 and 10 users participated in phase 3. The i-MoMCARE app comprises five core features: (1) clinical decision support system (CDSS), (2) electronic medical record (EMR) system, (3) educational resources, (4) digital worklog sorting and (5) offline functionality. Users reported that the CDSS improved consistency of care and boosted clinical confidence while the EMR enhanced data accuracy and retrieval efficiency. Educational resources were valued for supporting self-learning and community education. Meanwhile, worklog sorting improved time management and manpower planning, whereas offline functionality helped to ensure continued usage by mitigating connectivity issues. In addition, participants recognised the app’s potential to extend beyond clinical functions, highlighting its role in streamlining administrative tasks such as inventory tracking and automated reporting.

**Conclusions:**

This study demonstrates the value of CDSS and EMR systems in supporting VHSGs and health centre staff in their daily operations. Supplementing these with educational and administrative features further increases the app’s utility, positioning it as a comprehensive tool for advancing the delivery of MCH services in resource-limited settings.

**WHAT IS ALREADY KNOWN ON THIS TOPIC**

- Mobile health (mHealth) apps have the potential to support village health support groups’ (VHSGs) work performance, but adoption remains low due to poor usability and limited contextual relevance.

**WHAT THIS STUDY ADDS**

- A participatory approach with meaningful end-user involvement is critical to ensure that the mHealth app developed is usable and contextually relevant.
- This study demonstrates the added value of integrating clinical decision support systems and real-time electronic medical records to support the delivery of maternal and child health services.
- This study also highlights the potential of non-clinical features in mHealth apps to reduce the administrative burden of VHSGs.

**HOW THIS STUDY MIGHT AFFECT RESEARCH, PRACTICE OR POLICY**

- The findings provide actionable insights for designing scalable, user-centred mHealth solutions that are closely aligned with the actual needs of the end users.

## Introduction

Over the past decades, Cambodia has achieved steady progress in reducing maternal and infant mortality rates, mainly due to concerted efforts to expand the coverage^1^ and accessibility of maternal and child health (MCH) services.^2 3^ However, significant disparities remain, with rural areas continuing to experience disproportionately higher mortality rates compared with urban settings.^4^ These inequities are driven by persistent challenges, including limited healthcare services, inadequate infrastructure, and health workforce shortages in remote communities.^5 6^

To strengthen the capacity of the health system in addressing MCH service gaps in rural areas, task-sharing with community health workers (CHWs)—locally known as village health support groups (VHSGs)—has emerged as a practical and effective strategy.^7 8^ VHSGs are community-based volunteers who are recruited within the villages where they reside and receive basic training to deliver essential primary healthcare services at the community level. Their responsibilities span various preventive, promotive, and basic curative tasks, including managing paediatric conditions,^9^ immunisation,^10^ antenatal care (ANC), delivery, postnatal care (PNC),^11^ and communicable and non-communicable disease prevention, screening, and treatment.^12 13^ Their primary role involves conducting household visits, providing basic counselling, and referring patients to the nearest health centre when more advanced clinical care is required. However, referral processes are mainly manual and rely on personal communication—typically through phone calls or physical referral booklets—to link patients with public health facilities. This paper-based and person-dependent health information system often leads to delays, incomplete information transfer and limited follow-ups, highlighting the need for a digital solution to streamline coordination and continuity of care.

Given the breadth of their responsibilities and the often challenging environments in which they operate,^14^ VHSGs may benefit from mobile health applications (mHealth apps)—digital tools designed to support their work performance in the delivery of healthcare services.^15^ In recent years, the development and deployment of mHealth interventions have accelerated globally,^16^ driven by increased mobile device ownership and expanding network infrastructure, including in rural areas.^17^ These technologies have been particularly impactful in low- and middle-income countries (LMICs), where they support a range of functions such as clinical decision support,^18^ improved health information systems,^19^ behaviour change interventions^20^ and enhanced communication between health workers and patients.^21^ For end users like VHSGs, mHealth apps also have the potential to improve clinical accuracy,^22^ facilitate adherence to treatment protocols^23^ and provide upskilling opportunities^24^—ultimately enhancing their work performance.

However, low engagement with mHealth apps remains a critical barrier to realising their full potential, particularly where consistent use is essential for long-term impact.^25 26^ Poor usability, limited contextual relevance and lack of alignment with end-user workflows are common factors contributing to reduced adoption and sustained use.^27^ Increasingly, evidence suggests that involving end users in the design process can lead to higher levels of ownership, empowerment and long-term engagement.^28 29^ Therefore, active participation may foster a sense of shared purpose and ensure that the resulting solution is grounded in the realities of end users’ daily experiences.

In light of this context, the present study adopts a co-design approach to meaningfully engage end users—specifically VHSGs and health centre staff—in creating an mHealth app tailored to their needs.^30^ By incorporating end users’ perspectives throughout the design process, the development team can better align functionality, interface and workflows with the actual needs and preferences. This user-centred approach enhances the app’s relevance and usability, potentially strengthening the likelihood of sustained engagement and integration into routine healthcare practices.

## Materials and methods

### Setting

This study was part of a broader research initiative aimed at enhancing MCH service delivery in Cambodia through the development, implementation and evaluation of an mHealth app, i-MoMCARE.^31^ Building on insights from our previous studies, which identified key barriers to the performance and engagement of VHSGs,^32 33^ the present study focused on the design and development of the i-MoMCARE app to overcome some of these work challenges.

### Study design

The co-design strategy developed by Sanders & Stappers was used to guide the design of the i-MoMCARE app.^30^ It incorporates qualitative techniques within the rapid prototyping process (RPP). RPP comprises three phases. In this study, the approach was used to gather input from end users (VHSGs and health centre staff) to quickly design app prototypes.^34^ It also allows developers to rapidly evaluate and revise the prototypes based on end users’ feedback, resulting in a high-quality and functional final product.^35^

### Rapid prototyping process

#### Phase 1: Identification of potential app features (March–May 2024)

In phase 1, stakeholders were purposively engaged, including representatives from the Ministry of Health (MoH), the National Maternal and Child Health Centre (NMCHC), the University of Health Sciences (UHS) and the software development team. Stakeholders were presented with a comprehensive list of end-user needs that directly impacted the work performance and engagement of VHSGs and health centre staff in the delivery of MCH services. The list was developed based on findings from a systematic review and qualitative analyses previously conducted by the research team.^32 33^ Through collaborative discussions, stakeholders identified and proposed various app features to address end-user needs. The needs and corresponding stakeholder-proposed features are summarised in online supplemental file 1.

10.1136/bmjdh-2026-000074.supp1 Supplementary data

#### Phase 2: Prioritisation of app features for initial development (May 2024)

In phase 2, all stakeholders from phase 1 and researchers collaboratively prioritised key app features for initial development, recognising that it was not feasible to implement all the proposed app features simultaneously. To guide the selection of app features for initial development, a feasibility–impact approach was adopted. In phase 2, stakeholders and researchers collaboratively reviewed all proposed features identified in phase 1 (online supplemental file 1). Each feature was assessed and ranked based on its potential impact on community health outcomes, relevance to identified user needs, and feasibility of implementation within existing project resources and time constraints. Emphasising collaborative decision-making, this process provided an open forum for stakeholders and the study team to share insights, express differing perspectives, and collectively determine five essential app features for initial development (highlighted red boxes in online supplemental file 1). This participatory approach helped to align development efforts with the real-world needs of end users.

#### Phase 3: Prototype development, usability testing and feedback from end users (May–June 2024)

**Figure 1:**
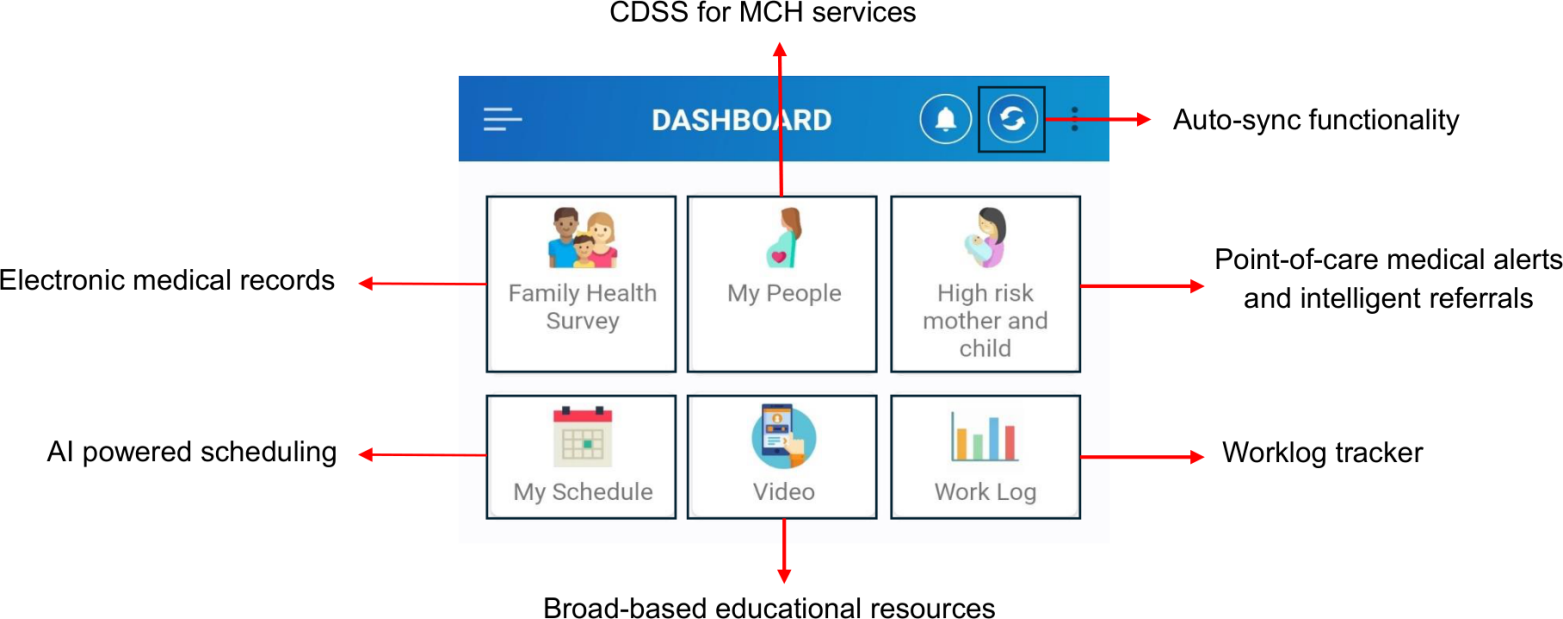
Snapshot of i-MoMCARE (app version) for VHSGs. CDSS, clinical decision support system; MCH, maternal and child health; VHSGs, village health support groups.

**Figure 2:**
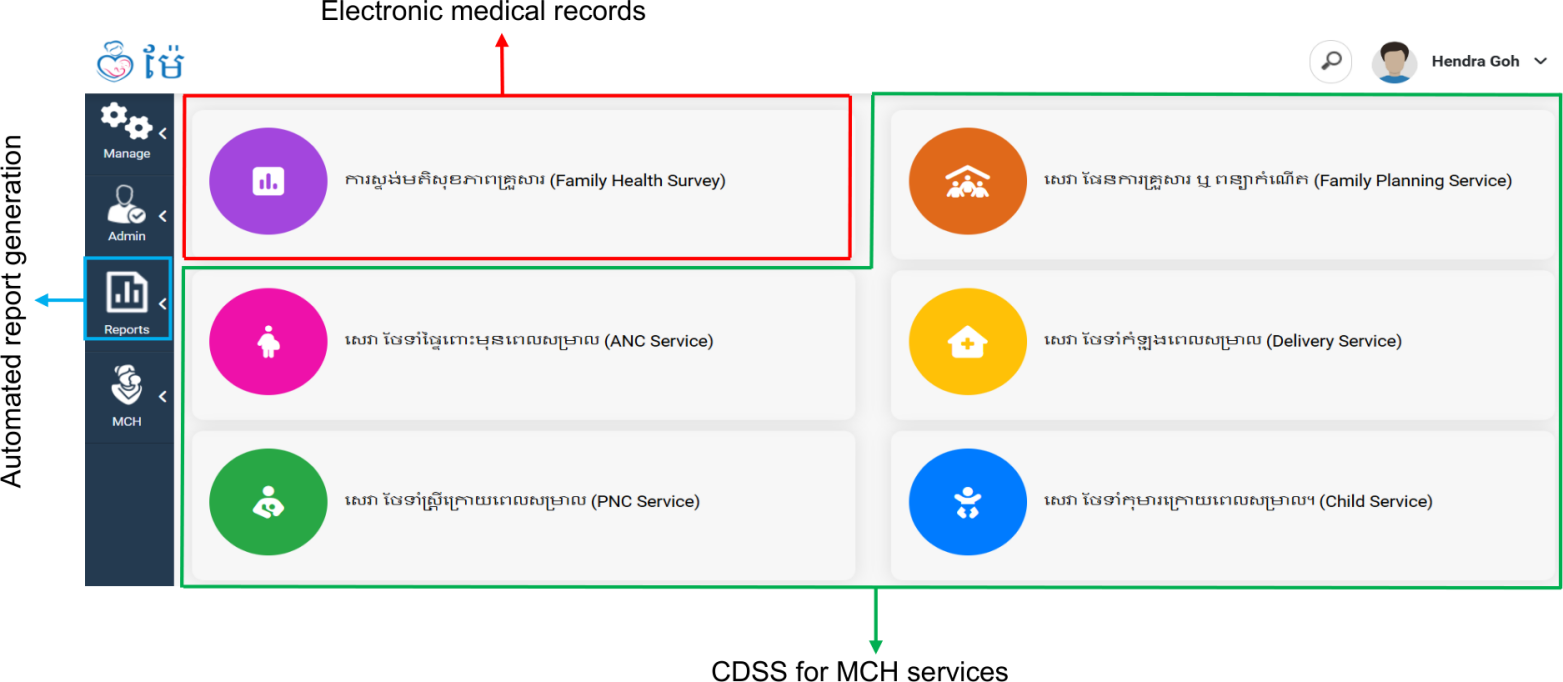
Snapshot of i-MoMCARE (web version) for health centre staff. ANC, antenatal care; CDSS, clinical decision support system; MCH, maternal and child health; PNC, postnatal care.

In phase 3, the study team conducted a technical validation process to assess the suitability of app features proposed for initial development, ensuring their alignment with technical capabilities. The software development team then proceeded to build the working version of the app. This involved the actual coding and configuration to create the i-MoMCARE app, incorporating features outlined in the co-design specifications (figures 1 and 2).

When the prototype had been developed, 10 VHSGs and health centre staff were purposively selected to participate in a 2-hour digital training session covering mobile phone and computer operation, troubleshooting, and detailed app usage. Two versions of the system were developed: a mobile app for VHSGs and a web-based platform for health centre staff. The web version is accessed via desktop with secure login, while the Android mobile app enables VHSGs’ community work. This design reflects device availability and affordability in LMICs, ensuring accessibility and alignment with user contexts and workflows. This was followed by a 20 min free-and-easy session, allowing participants to explore the app independently. Subsequently, the trainers presented various patient case scenarios and asked the participants to use the app to simulate care provision and medical record entry. At the end of phase 3, feedback from all participants was collected through rapid qualitative interviews, serving as a valuable reference for the study team to refine the app and address potential issues. App features and design enhancements were implemented subsequently based on the feedback. Three trained interviewers (HG, CD and DK) conducted the interviews, each lasting between 15 and 30 min, and all sessions were audio-recorded following verbal consent. In total, 10 rapid qualitative interviews were conducted. The duration of interviews ranged from 15 to 30 min.

### Data analysis

A topic guide was developed through a structured, evidence-informed process. First, findings from our preceding systematic review and qualitative study were synthesised to identify recurrent barriers, facilitators and workflow challenges experienced by VHSGs and health centre staff.^32 33^ These themes served as the preliminary conceptual foundation. Second, a panel of subject-matter experts—including clinicians, public health researchers, digital health specialists and programme implementers—reviewed these preliminary domains to ensure clinical relevance, contextual appropriateness and alignment with MCH service delivery processes in Cambodia. The topic guide was then iteratively refined through multiple rounds of discussions within the research team, focusing on clarity and feasibility for field implementation. The discussions resulted in two major topics focusing on the perceptions of specific app features within the i-MoMCARE app and suggestions for potential improvements. Rapid qualitative analysis was employed to analyse end users’ feedback, involving the development of neutral analytic domains a priori, aligned with each interview question. A standardised summary template was constructed based on these domains to facilitate consistent data organisation. The template was pre-tested for clarity and usability before being independently applied across all interviews by two analysts (HG and YFL). Transcripts were coded, and the resulting themes were thematically presented to identify key insights and patterns.^36^ Coding was conducted using NVivo V.15. Fieldnotes were also recorded during each interview. The study followed the Consolidated Criteria for Reporting Qualitative Research checklist to ensure methodological rigour and transparency (online supplemental file 2).

10.1136/bmjdh-2026-000074.supp2 Supplementary data

### Patient and public involvement

Patients and/or the public were not involved in the design, conduct, reporting or dissemination plans of this research.

## Results

### Demographics of participants

A total of 16 stakeholders participated in phases 1 and 2, comprising five representatives from the MoH and NMCHC, six academic researchers and five software developers. The group brought together diverse expertise, including medicine, midwifery, pharmacy, public health, data science and engineering. In phase 3, 10 end users (6 health centre staff and 4 VHSGs) participated in the usability testing and qualitative interviews. Across all three phases, 26 individuals participated, including 16 females (61.5%) and 10 males (38.5%). Participants ranged in age from 28 to 66 years, representing a broad spectrum of professional experience and demographic backgrounds. No participants dropped out from the study.

### App features

At the end of phase 2, stakeholders prioritised five key app features for initial development. Table 1 outlines the functionality and purpose of each app feature in detail. Following development, these app features were tested by end users during phase 3, with participants providing feedback on their usability and relevance. Table 2 summarises participants’ perceptions of each app feature.

**Table 1:** i-MoMCARE app features

End user needs	Features	Description
Skill and confidence building	Clinical decision support system (CDSS)	CDSS is developed specifically targeting the five core maternal and child services: Family planning (FP) services: To guide the delivery of FP services including condom use, combined oral contraceptives, progesterone-only pill, emergency contraception, contraceptive injection, contraceptive implant and intrauterine device. Antenatal care (ANC) services: To guide the delivery of ANC services including general ANC check-up, identification of danger signs, ANC counselling, blood tests, medications and vaccination Delivery services: To guide the delivery of parturition services including labour stage, delivery stage, breastfeeding counselling, immediate newborn care, medications and vaccination. Postnatal care (PNC) services: To guide the delivery of PNC services including general PNC check-up, identification of danger signs, PNC counselling, follow-up newborn care, medications and vaccination. Child health services: To guide the delivery of child health services including well baby visits and childhood immunisation. Point-of-care medical alerts are integrated into the CDSS to automatically notify users of high-risk patients during service delivery. These alerts support timely clinical decision-making by highlighting critical conditions or risk factors.
Health data management system	Electronic medical records (EMRs)	The EMR system is designed to function as a secure repository for health data. It captures: Personal information for each patient, including detailed medical history and current medications. Past medical records, systematically transferred from health centres’ pen-and-paper booklets, to build a centralised health profile.
Self-educational improvement and community education	Broad-based educational resources	Multimedia resources for self-improvement and community education are provided and periodically updated. The resources are organised into two categories: Self-learning resources for healthcare professionals, covering various health topics through videos to support continuous professional development. Community education resources focused on promoting healthy lifestyles, basic hygiene, breastfeeding counselling, and newborn care, delivered exclusively through videos to enhance accessibility and comprehension among community members.
Workload management	Digital worklog sorting	An automated workload distribution tool to help prioritise tasks and manage workload. Powered by an advanced algorithm, the platform sorts the cases that healthcare professionals need to see each day according to the severity of symptoms.
Stable internet connectivity	Offline functionality	Robust offline functionality allows users to access and use the platform without interruption during periods of limited or no internet connectivity. Data entered offline are automatically synchronised with the cloud system once the device reconnects to the internet.

**Table 2:** Perception of specific features of the i-MoMCARE app

Features	Themes	Illustrative codes
Clinical decision support system	Standardising clinical care through real-time guidance improves patient outcomes and provider confidence	*“The ANC and PNC features help women in my village achieve better safety before and after childbirth. In ANC, when we conduct our visit, we will be guided to perform a series of prenatal check to ensure that the mom is okay. In PNC, the feature will also guide us to check on the mom and child after birth to identify any complications. It makes me to be more confident and consistent when giving care.”(VHSG, F, 48*) *“For example, when the patient comes in for delivery, the delivery form in the app will show me exactly what parameters I need to measure step-by-step. This ensures that I provide standardised care to all patients. This way I also feel more assured as everything is done according to guidelines.”(HC staff, F, 34*)
	Preventing negligent or inconsistent entries through automated logic checks	*“MyPeople has a function on child vaccination. From the table, vaccines are colour coded, meaning completed vaccines will be marked in green and missed vaccines will be highlighted in red. I can tell which vaccination is missed at a glance and I can ask the mother to bring the child to receive the vaccines accordingly. It also helps me to not make any negligence.”(VHSG, F, 39*) *"In MyPeople, if we enter incorrect or nonsensical data—like recording a newborn’s weight as 2500kg instead of 2500g—the system won’t let us submit it. It’s really helpful because it prevents mistakes and keeps us accountable.”(HC staff, M, 41*)
	Danger sign identification promotes early detection and timely intervention	*“I think [the] danger signs of pregnant women is the most useful features to me because we need to follow up these women more closely. Also, health centre staff can also take note that they are of high risk and provide care more regularly.”(VHSG, F, 26*)
	Pop-up notifications and error prevention	*“Whenever abnormal clinical parameters are entered, the system displays a red alert message, prompting me to review the data for accuracy. For instance, if a diastolic pressure of 220 mmHg is recorded, the system immediately flags this as an unusually high value, reminding me to verify the entry before proceeding.”(HC staff, F, 28*)
Electronic patient registration and tracking	Digital registry that is easily accessible and secure	*“With electronic medical record, it is also easier for me to retrieve the patient details, just by typing in the phone! Also, I can just edit it, and it will still look clean. Previously, I had to write it down manually in booklets and additional corrections can be really messy”(VHSG, M, 52*) *“Before, I have to carry paper [to record patient data] and it might be raining or worse still, I might even lose the booklet. But now data storage becomes safer as we only need to use the work phone to add and access these data.”(VHSG, F, 48*)
Broad-based educational resources	Community health promotion videos: valuable visual aids for health literacy	*“We can educate the women who are sometimes illiterate to know about pregnancy care. As it is visual, it is very easy for them to understand. So, they [can] breastfeed their children for a longer period. [We can also show them videos that] breastfeeding does not affect the health of women and children, and it can reduce the risk of child infections.”(VHSG, F, 39*)
	Self-directed learning materials: enabling flexible and pace-adjusted skill development	*“While MyPeople [name for the CDSS in i-MoMCARE] is indeed useful, I think the problem is I am not well equipped to begin with. Therefore, the specially curated modules such as topics on integrated childhood illness management allow me to upskill myself at a pace I am comfortable with. I [tailor] the learning process according to my level of understanding. It is important to continue improving ourselves to better care for the community.”(VHSG, F, 26*) *“I like the materials because two separate sets are prepared, one set which comprises of only videos to educate the women in the community whereas another set for us to educate ourselves. At workplace, we hardly have any training or talks. With this, I can access it anytime and anywhere… Oh, the mix of articles, videos and quizzes are good too, so it is not that boring when I am studying on my own!”(VHSG, M, 52*)
Digital worklog sorting	Enhanced prioritisation and workload management through automated triage	*“I appreciate the feature for automated workload sorting, which organises my daily caseload based on urgency. It appears to consider factors such as the presence of high-risk symptoms and the number of missed appointments. This helps me prioritise and attend to the most critical patients at the beginning of the day.”(VHSG, M, 52)* *“This feature has improved how we manage limited manpower. I advised the VHSGs that it is acceptable if they cannot see all patients in one day, as long as they prioritise those with more urgent needs and defer less critical cases to a later time.”(HC Staff, F, 25*)
Offline functionality	Offline functionality supports service continuity in low-connectivity settings	*“The app’s ability to function without internet connectivity makes it particularly suitable for rural areas, where access to the internet can be intermittent or where electricity outages may occur.”(HC Staff, F, 32*) *"In some households, internet access is unavailable. Despite this, I’m still able to retrieve patient records and input clinical information. However, I do need to remember to manually refresh the app to ensure that newly entered data are synced when connectivity is restored.”(VHSG, F, 39*)
Administrative functionalities	Potential of administrative functionalities to streamline daily operation	*“I think we [should] think beyond the clinical features of the app and extend it to administrative tasks such as automated report generation. Currently, we need to do [manual] counting to prepare for the monthly report. So, it may be good if we can just click [in the app] to generate the report automatically.”(HC staff, M, 41*) *“One of the issues we sometimes face at the health centre is running out of medications—not because the central supply didn’t send them, but simply because we forgot to restock in time. That’s why I hope the app can track stock levels and give us reminders to replenish early.”(HC Staff, F, 32*)

#### Feature 1: Clinical decision support system

In phase 2, stakeholders concurred that the lack of working knowledge and confidence in treating patients was the most fundamental barrier preventing VHSGs and health centre staff from delivering MCH services. As such, clinical decision support system (CDSS) was developed as an app feature specifically targeting the five core MCH services: family planning, ANC, delivery care, PNC and child health services. It provided end users with stepwise guidance in physical examination, patient counselling, identification of danger signs, and auxiliary services such as ordering blood tests and medication. Following usability testing in phase 3, participants emphasised that CDSS-structured workflows enhanced the consistency of care across different MCH services. The step-by-step guidance on patient care ensured that healthcare providers conducted comprehensive and standardised health assessments. At the same time, it also boosted their confidence, particularly during clinical uncertainties.

This app feature was also enhanced with the point-of-care medical alerts to provide timely notifications about critical health conditions, ensuring that high-risk cases were not missed. In routine practice, danger signs were identified through fragmented paper checklists and providers’ clinical judgement, leading to variability in detection. By digitising this process, the app supports systematic danger-sign identification, enabling earlier recognition and timely intervention for high-risk cases. Most participants valued this app feature as particularly relevant for high-risk pregnancies. In particular, VHSGs noted that it helped them monitor at-risk women more closely and prompted timely referrals to health centres when necessary. Supplemented by pop-up notifications, it provided immediate alerts when abnormal or erroneous data were recorded.

During the development stage, the CDSS underwent an internal validation process to ensure its logical accuracy, consistency and reliability. The embedded clinical algorithms and decision trees were systematically mapped to the Cambodian MCH guidelines to ensure full alignment with existing care standards. Expert review sessions were conducted with clinicians and specialists from the NMCHC to verify the correctness of clinical logic, diagnostic pathways and alert functions. In addition, simulated case scenarios were used during the rapid prototyping stages to identify and resolve potential computational or logic errors. This iterative testing process helped refine the accuracy of the CDSS outputs prior to field deployment. A more comprehensive validation—including assessment of predictive accuracy, usability and clinical reliability—was reported in another separate study (publication in progress).

#### Feature 2: Electronic medical records

In phase 2, stakeholders noted the absence of a formal electronic medical record (EMR) system in Cambodia, resulting in the continued reliance on paper-based methods for clinical data management. In response, a localised EMR system was developed, tailored to the workflows of the Cambodian primary healthcare context. The EMR component of the i-MoMCARE platform was developed using OpenMRS, an open-source EMR framework widely adopted in LMICs. OpenMRS was selected due to its modular architecture, flexibility for customisation and strong alignment with digital health standards.^37^ Building on this platform, we configured an EMR that consolidates essential administrative and clinical information under each patient’s profile, including demographic data, obstetric history, antenatal and postnatal care records, immunisation status, diagnoses and follow-up appointments.

This transition from paper-based records to an electronic medical registry marked a significant improvement in efficiency and data security. During qualitative interviews in phase 3, participants appreciated the ability to retrieve up-to-date patient details and reduce the clutter associated with manual record-keeping. The secure digital storage of patient information also addressed logistical challenges, such as weather damage or loss of physical booklets, ensuring greater reliability in data management. A built-in cross-checking mechanism was added to the EMR to safeguard against clinical negligence and reduce oversight. For instance, data validation checks prevented input errors, such as rejecting implausible values like an excessively high birth weight. Participants found this functionality valuable in maintaining accountability and ensuring the accuracy of patient records.

The system also employs role-based access control, ensuring that VHSGs and health centre staff access only the information relevant to their scope of practice. Data captured through the EMR serve as the foundational patient record on which the CDSS operates, enabling more contextually informed decision-making at the point of care. While the EMR currently functions within the primary care setting, interoperability with higher-level referral facilities is planned for future phases of system integration.

Finally, recognising that the EMR system handles sensitive patient data, stringent data security and privacy measures were embedded throughout the development process. All data are stored on encrypted servers managed by an authorised technological partner—Argusoft and overseen by staff from the state university—UHS, Cambodia, with access restricted through role-based authentication and password protection. Data transmission between devices and servers is secured using Secure Socket Layer encryption, and sensitive identifiers are anonymised where appropriate. Regular system audits, data backups, and integrity checks are conducted to ensure the confidentiality and reliability of stored information. The app was designed in alignment with the Cambodian MoH’s digital health governance principles and internationally recognised standards for data protection in mHealth apps.^38^

#### Feature 3: Broad-based educational resources

While the CDSS may provide immediate support in navigating clinical uncertainties, stakeholders in phase 2 unanimously emphasised the urgency to upskill the existing workforce as a sustainable, long-term solution to this challenge. To aid their professional development, the app was developed to include in-app references and educational materials. It enabled the upload of seminars, lecture notes, and reading materials to facilitate didactic learning. A quiz section was also added, organised by health topics, to allow users to test their knowledge after viewing the learning materials. The inclusion of structured learning modules allowed healthcare providers to enhance their clinical knowledge at their own pace. As quoted in phase 3, participants appreciated the flexibility to tailor their learning based on their level of understanding.

Recognising the low levels of health literacy within the community, some stakeholders recommended incorporating a dedicated section within the app specifically focused on community education. This section mainly comprises short video clips specially curated to educate patients during their visits as part of outreach efforts. Integrating visual learning materials was perceived as beneficial for improving health literacy in communities with low literacy rates. During qualitative interviews, participants emphasised that videos were effective in educating women on topics that require demonstration, such as breastfeeding. The visual format made complex health messages more accessible, encouraging behaviour change.

#### Feature 4: Digital worklog sorting

Stakeholders identified high workload and its associated stress as longstanding challenges within the Cambodian health system, particularly highlighted during discussions in phase 2. To help address this issue, a digital workload sorting feature was developed, designed to assist users in prioritising patients based on clinical urgency. This app feature employs a rule-based algorithm to automate patient triaging, taking into account factors such as symptom severity and the frequency of missed appointments. This enabled healthcare providers to focus on high-risk cases first, supporting more efficient time and resource management. Qualitative insights from participants in phase 3 further reinforced the perceived value of this app feature in enhancing time and resource management, particularly during periods of limited manpower.

#### Feature 5: Offline functionality

At a broader systems level, infrastructure challenges—particularly unreliable internet connectivity—were another key concern raised in phase 2 stakeholder consultations. In response, the app was subsequently developed with robust offline functionality to ensure continuity of service delivery in low-connectivity settings. This app feature enabled end users to enter and access patient data without an active internet connection, with automatic synchronisation occurring once connectivity was restored. Further illuminating the advantages of the offline functionality, participants noted the app’s suitability for rural settings where internet connectivity and electricity supply are often unreliable. This helped to safeguard the integrity and continuity of health records regardless of network availability, offering a resilient solution to infrastructure-related limitations.

### Suggested improvements and implemented changes

Feedback for improvement from VHSGs and health centre staff was collected at the end of phase 3 (table 3). The majority of participants identified CDSS and EMR as the core app features most integral to their daily operations, citing their potential to improve workflows compared with existing practices. As a result, suggestions related to the CDSS and EMR primarily focused on optimising existing functionalities rather than making foundational changes. Recommendations included technical enhancements such as adding new data entry fields, adding previously omitted patient information, and refining interface elements. These user-driven suggestions informed iterative updates to enhance end users’ experience and system efficiency.

**Table 3:** Suggestions for the i-MoMCARE app and solutions

Feedback	Solutions	Change implemented
Clinical decision support system–family planning services		
Expected date of removal for contraceptive injection, contraceptive implant and intrauterine device are missing	To enable editing of expected date of removal for contraceptive injection, contraceptive implant and intrauterine device	Yes, after phase 2
clinical decision support system–antenatal care services		
Last menstrual period date cannot be edited to reflect the most recent or updated information	To enable editing of last menstrual period date	Yes, after phase 3
Estimated date of delivery is missing	To automate the calculation of estimated delivery date	Yes, after phase 3
Blood test results of the patient’s husband are missing	To introduce a section within the patient’s profile to record and store her husband’s blood test results	No, possibly in future builds
Clinical decision support system–delivery services		
High-risk signs and symptoms identified from antenatal care records are not shown in delivery records	To implement an automatic transfer of high risk signs and symptoms from antenatal records	Yes, after phase 3
Missing items in breastfeeding counselling and immediate newborn care sections	To update breastfeeding counselling and immediate newborn care sections	Yes, after phase 2
Clinical decision support system–postnatal care services		
Delivery records are not shown in postnatal care records	To implement an automatic transfer of delivery records	Yes, after phase 3
Maternal and child death records are missing but required for reporting to the Ministry of Health	To implement a separate section for documenting maternal and child death records	Yes, after phase 3
Postnatal vaccination counselling is missing	To implement a separate section on postnatal vaccination and enable previous vaccination records	Yes, after phase 3
Clinical Decision Support System–Child health services		
Past childhood immunisations given during postnatal care visits are missing	To implement an automatic transfer of childhood immunisation from postnatal records	Yes, after phase 3
Immunisations that are due but not yet administered are not reflected	To introduce a red colour-coding scheme to label missed immunisations for immediate identification	Yes, after phase 2
Difficult to distinguish between immunisations that have already been administered and those scheduled for future dates	To introduce a green colour-coding scheme to label immunisations that have already been administered	Yes, after phase 2
The system does not support scheduling of next appointment for upcoming immunisation visits	To create a calendar function for scheduling next immunisation appointment	Yes, after phase 3
Immunisation counselling is missing	To implement a text-based section to document immunisation counselling	Yes, after phase 3
Electronic medical records		
Current search function only allows queries by name and date of birth.	To allow identification of patient based on phone number and location	Yes, after phase 2
For services that require more than one visit, previous records are not automatically shown	To enable linkage of records across multiple visits for services that require follow-up	Yes, after phase 3
Occupations of the patient and family members are missing	To collect occupations of the patient and family members during registration	Yes, after phase 2
Healthcare assistance card information is not collected	To create a QR code scanning function that automatically populates the healthcare assistance card information field	Yes, after phase 2
Broad-based educational resources		
Preference for greater variety of self-learning resources	To include lecture notes and quizzes arranged according to modules	Yes, after phase 3
Preference for educational games to increase clinical knowledge and engagement	To develop educational games in the game centre	No, possibly in future builds
Digital worklog sorting		
High-risk cases that are not seen today are not carried forward to the next day	To implement an automatic transfer of uncompleted high risk cases to the next day	Yes, after phase 3
Other functionalities		
Importance of inventory management and planning such as vaccinations, medications and contraceptives	To implement a stock monitoring system with the capability to generate monthly stock usage reports	Yes, after phase 3
Monthly health centre performance reports are currently prepared manually	To generate automated health centre performance reports	Yes, after phase 3
Referral of patients to stepped up care is currently done manually over telephone and/or email	To allow in-app patient referral	Yes, after phase 3
Desire for secured intraprofessional communication	To allow in-app messaging function	No, possibly in future builds

Meanwhile, other app features like broad-based educational resources and digital worklog sorting were viewed as complementary app features, serving primarily to support and enhance the usability of the mHealth app. In particular, VHSGs and health centre staff suggested expanding the educational content beyond static resources such as lecture videos. They recommended including interactive components, such as quizzes and games, to create a more engaging learning experience. While participants appreciated the availability of static materials, they expressed a preference for interactive formats, noting that such app features could enhance motivation and retention. In response, quizzes were incorporated into the app by the end of phase 3. Although complete gamification was yet to be implemented, the study team recognised its potential to nudge end users’ engagement and has initiated plans to develop a dedicated game-based learning module.

By the end of phase 3, VHSGs and health centre staff proposed several enhancements that extended beyond the app’s initial clinical focus (see table 3 for other functionalities and table 2 for administrative functionalities for quotes). While the app was initially designed to support the end users’ work performance and engagement, participants emphasised its potential to streamline administrative tasks. One key suggestion was incorporating an inventory management system to track supplies such as vaccines, medications and contraceptives. As consumables usage has to be systematically recorded, a digital stocklist could replace manual counting and help ensure timely replenishment. Additionally, participants recommended the inclusion of an automated reporting feature to generate monthly reports required by the MoH. This app feature would improve efficiency and reduce errors commonly associated with manual report preparation. Participants also suggested enhancing the in-app patient referral system to replace current phone-based methods for referring patients to tertiary care facilities, and a secure in-app messaging platform to facilitate professional communication regarding patient care. These proposed app features reflected a broader vision for the app as an integrated tool supporting clinical and administrative workflows.

## Discussion

As part of the i-MoMCARE trial,^31^ this study used the co-design approach to develop an mHealth app as a practical job aid for supporting the work performance and engagement of staff delivering MCH services in rural Cambodia.^30^ It also explored end users’ perspectives to inform user-centred app refinement. Employing a stakeholder participatory approach provided valuable insights into end users’ experiences when engaging with the app, allowing for a more nuanced understanding of how the mHealth solution could be optimised for real-world application.

Notably, several VHSGs emphasised limitations in their current knowledge and skillsets, which hindered their confidence in carrying out their responsibilities. Findings from our previous study indicated that this challenge primarily stemmed from scarce access to structured training programmes and professional development opportunities, leaving them inadequately prepared for work.^33^ As a result, VHSGs need a supportive tool to guide them through patient care, emphasising the importance of clinical accuracy and safety. To address this need, a CDSS incorporating elements of personalised medicine and predictive analytics was developed. At its core, the CDSS incorporates existing national MCH guidelines to provide end users with step-by-step guidance and structured prompts during patient consultations. Aligned with the growing emphasis on personalised care,^39^ the system supports decision-making by leveraging unique patient data from the EMR, including health status and lifestyle factors. By incorporating these variables that are often overlooked, the CDSS enables healthcare providers to develop more precise and personalised care plans, thereby reducing the risk of adverse outcomes and improving the quality of care delivered at the point of service.^40^ Additionally, the inclusion of predictive analytics enables the system to identify potential health risks and forecast disease progression, supporting early intervention and preventive strategies to enhance overall patient outcomes.^41^

Our study also highlights the role of a real-time data repository in supporting the CDSS for integrated care delivery. Currently, patient medical histories are recorded manually using pen and paper—a method that is often inefficient, prone to loss or damage, and difficult to access across different service points.^42^ To address this challenge, we developed an EMR system that consolidates all administrative and clinical information within each patient’s profile in real time, thereby creating a unified repository of patient data. While the CDSS facilitates evidence-based clinical decision-making, the EMR component supplies essential contextual information—such as current medications, diagnostic history and recent laboratory results—enabling healthcare providers to make well-informed decisions at the point of care.^43^ Through analytically mining the data stored within the EMR, the CDSS can generate better clinical knowledge that is dynamically tailored to individual patient profiles. This knowledge analytics synergy ensures that clinical actions are not only aligned with standardised national guidelines but also relevant within the context of the patient’s unique clinical status.^44^

Even when CDSS offers critical support in guiding end users through clinical uncertainties by compensating for gaps in knowledge and skills,^45^ participants acknowledged that such app feature serves only as a temporary aid. They emphasised the importance of professional development for long-term sustainability. Participants also expressed a strong sense of accountability and the need to take ownership of their learning journeys, highlighting the role of intrinsic motivation in sustaining professional growth.^46^ In our study, participants expressed a preference for interactive learning tools over static materials, reporting that quizzes and gamified modules might keep them more engaged in the learning process. This feedback aligns with evidence that purely lecture-based approaches often lack engagement,^47^ whereas interactive learning resources can promote active recall and reward progress, which reinforces learning and sustains user engagement.^48^ As a result, a blended learning approach comprising both static and interactive modalities was adopted in the design of educational materials to foster greater learning independence among end users.^49^ This app-based blended learning approach is especially relevant in resource-limited settings, where mHealth apps can transcend geographic barriers to deliver interactive training content and keep distributed healthcare providers engaged in continuous learning.^50^

Finally, the expanded use of mHealth apps to support administrative tasks underscores the evolving needs of VHSGs and health centre staff and the value of multifunctional digital tools in resource-limited settings. Participants in this study see considerable value in expanding the app’s functionality beyond its original clinical focus to address key administrative challenges. Suggestions, such as integrating inventory management, automated reporting, digital referrals and secure communication, reflect the need for a more holistic digital solution supporting operational efficiency.^51^ These proposed enhancements indicate that administrative workload—often performed manually—poses a significant mental burden.^52^ By streamlining these tasks, the app can help reduce cognitive load, improve data accuracy and free up time for direct patient care.^51 53^

### Limitations

A key limitation of this study is that end users (VHSGs and health centre staff) were only engaged in phase 3, rather than throughout all stages of the co-design process. Earlier involvement of end users may have contributed to the development of a more contextually relevant and user-centred prototype. However, in the absence of any pre-existing mHealth apps for MCH in the Cambodian context, it was anticipated that participants might find it challenging to conceptualise or propose app features from the ground up. As such, end users were included primarily in the testing and feedback phase, after the foundational design of the prototype had been established empirically. To ensure that end users’ perspectives were still reflected in the earlier design phases, staff from the NMCHC—many of whom previously served as health centre staff and had extensive experience working with VHSGs—were actively involved in Phases 1 and 2. Their practical experience and familiarity with field-level service delivery helped ensure the prototype’s relevance and alignment with real-world needs.

In addition, despite receiving a 2-hour digital training session and a subsequent hands-on exploration period, participants may not have been fully familiar with all the app functionalities during phase 3. As this was their first encounter with a comprehensive digital MCH platform, limited familiarity may have influenced their interaction with certain app features and affected their subsequent responses during the rapid qualitative interviews.

Lastly, as the i-MoMCARE app is the first digital tool introduced to support MCH care in Cambodia, participants often focused on its improvements relative to current paper-based practices. Consequently, negative feedback was less prominent in the interviews. This limitation requires refinement and should be further explored in future studies.

## Conclusions

This study employed a stakeholder participatory approach to develop an mHealth app to improve work performance and engagement of community health workers and healthcare providers working in primary health settings in MCH services delivery. We also explored end users’ opinions during the development process, enabling iterative refinements to ensure the app was aligned with their needs. The study highlights the synergistic value of incorporating an EMR system with the CDSS to promote more integrated care. The combined functionalities, such as personalised medicine, predictive analytics and real-time data integration, are particularly valuable in settings with limited clinical capacity and underdeveloped health infrastructure. Moreover, a balanced mix of passive and interactive learning resources can promote end users’ engagement and foster continuous skill development. Extending the app’s functionality beyond clinical support to include administrative tasks further increases its utility and positions it as a comprehensive tool for routine use in primary healthcare delivery.

## Data Availability

Data are available on reasonable request.
