# Supplementary material for: Developing the i-MoMCARE application in Cambodia: an iterative co-design approach using rapid prototyping
Source: BMJ Digit Health Ai. 2026 Jun 10;2(1):e000074. doi: 10.1136/bmjdh-2026-000074 (PMC13528386; doi:10.1136/bmjdh-2026-000074)
Supplement: Supplementary file 1 [file bmjdh-2-1-bmjdh-2026-000074-s001.pdf]

Supplementary File 1. Collated end-user needs and proposed features

1. Personal level needs

- **Self-educational improvement:** Need for enhanced educational resources to improve their knowledge and clinical skills.
  Feature: *An interactive e-learning platform with access to video tutorials, webinars, and up-to-date medical literature. The platform includes quizzes and certifications to track progress and enhance clinical skills.*

- **Self-efficacy:** Need for mechanisms to strengthen their sense of self-efficacy in patient management.
  Feature: *A self-assessment and goal-setting module that enables Village Health Support Groups (VHSGs) to track their personal growth and achievements.*

- **Skill and confidence building:** Need for upskilling, clinical support, and measures to boost confidence in delivering healthcare services.
  Feature: *A clinical decision support system (CDSS) that guides the provision of core maternal and child health services.*

2. Interpersonal level needs

- **Family support:** Need for strategies to educate and gain support from family members, particularly in overcoming traditional gender role resistance.
  Feature: *Electronic educational resources and workshops aimed at family members, helping them understand and support the roles of VHSGs.*

- **Workplace relationships:** Need for a supportive work environment, including improved collegial relationships and reduced workplace competition.
  Feature: *Secured in-app chat to ease intra-professional communication and build collegial relationships.*

3. Organisational Level Needs

- **Adequate training:** Need for comprehensive on-the-job training and access to continuous professional development opportunities.
  Feature: *A comprehensive training dashboard with access to on-the-job training modules, webinars, and continuous professional development courses.*

- **Effective supervision:** Need for constructive feedback and guidance from supervisors to enhance job performance and professional growth.
  Feature: *A feedback and reporting system where supervisors can provide constructive feedback and guidance, alongside a professional development tracker.*

- **Resource availability:** Need for adequate resources, including medical supplies, infrastructure, and qualified personnel.

Feature: *A resource management portal that provides real-time updates on the availability of medical supplies and access to support services.*

- **Career growth:** Need for clear career progression pathways and opportunities for advancement.
  Feature: *A career planning tool that outlines clear progression pathways, including training requirements, opportunities for advancement, and potential career trajectories.*

- **Workload management:** Need for assistance in managing and prioritising workloads.
  Feature: *An automated workload distribution tool that helps prioritise tasks and manage workload.*

4. Community level needs

- **Community education:** Need for initiatives to improve community health literacy and correct misconceptions about healthcare.
  Feature: *A library of community-friendly educational materials, including pamphlets, videos, and interactive sessions designed to improve health literacy and correct misconceptions.*
- **Cultural sensitivity:** Need for guidance in navigating cultural norms and practices that impact healthcare delivery.
  Feature: *A cultural competence e-workshop that offers guidelines and best practices for navigating cultural norms and practices in healthcare delivery.*

- **Safety and security:** Need for protection and support against harassment and violence, particularly for female VHSGs.
  Feature: *A safety alert system that allows VHSGs to report incidents of harassment or violence and access resources for protection and support.*

5. Health system level needs

- **Health data management system:** Need for a secure and efficient health data management system to support the collection, storage, and retrieval of patient information across healthcare service points**.**
  Feature: *An electronic medical record that captures a patient's personal data and health information at the point of registration. Future medical records will be stored within the system, enabling easy retrieval and seamless sharing across health institutions to support continuity of care and coordinated service delivery.*
- **Financial stability:** Need for regular and adequate financial compensation, along with transparency in remuneration processes.
  Feature: *A transparent financial dashboard that provides clear information on compensation, benefits, and payment schedules.*

- **Policy and guidelines access:** Need for easy access to health policies and guidelines to ensure compliance with best practices.
  Feature: *A centralised repository of health policies, guidelines, and best practices, accessible at any time for reference and compliance.*

- **Stable internet connectivity:** Need for a stable and reliable internet connectivity to ensure seamless access to digital resources, real-time data entry and record retrieval.
  Feature: *The app should include offline functionality, allowing users to continue accessing essential features, entering data, and using critical tools even when internet connectivity is unstable or unavailable. Once a stable connection is re-established, the app should automatically synchronise the data, ensuring no information is lost.*

- **Professional recognition:** Need for recognition and respect within the broader health system to validate their roles and contributions.
  Feature: *The app may include a performance dashboard that display individual and team achievements (e.g., number of patients served, successful follow-ups) to supervisors. At the same time, award badges and certificates for milestones, trainings completed, or outstanding service, which can be shared within the health system.*
