# Supplementary material for: Developing the i-MoMCARE application in Cambodia: an iterative co-design approach using rapid prototyping
Source: BMJ Digit Health Ai. 2026 Jun 10;2(1):e000074. doi: 10.1136/bmjdh-2026-000074 (PMC13528386; doi:10.1136/bmjdh-2026-000074)
Supplement: Supplementary file 2 [file bmjdh-2-1-bmjdh-2026-000074-s002.pdf]

Consolidated criteria for reporting qualitative research (COREQ): 32-item checklist

Item number	Guide questions	Reported on
**Domain 1: Research team and reflexivity**		
Personal Characteristics		
1. Interviewer/facilitator	Which author/s conducted the interview or focus group?	Data collection in Methods section pg 6.
2. Credentials	What were the researcher's credentials? E.g. PhD, MD	MD, PhD, MPH.
3. Occupation	What was their occupation at the time of the study?	Academic faculty in the University.
4. Gender	Was the researcher male or female?	Males and females.
5. Experience and training	What experience or training did the researcher have?	Training in health services and public health.
Relationship with participants		
6. Relationship established	Was a relationship established prior to study commencement?	No relationship was established prior to study commencement.
7. Participant knowledge of the interviewer	What did the participants know about the researcher? e.g. personal goals, reasons for doing the research	Participants were aware of the primary purpose of the study (Data collection in Methods section pg 5).
8. Interviewer characteristics	What characteristics were reported about the inter viewer/facilitator? e.g. Bias, assumptions, reasons and interests in the research topic	Interviewer has extensive experience in qualitative research (Data collection in Methods section pg 6).
**Domain 2: study design**		
Theoretical framework		
9. Methodological orientation and Theory	What methodological orientation was stated to underpin the study? e.g. grounded theory, discourse analysis, ethnography, phenomenology, content analysis	Thematic analysis (Data analysis in Methods section pg 6-7).
Participant selection		

Goh H, *et al. BMJ Digit Health* 2026; 2:e000074. doi: 10.1136/bmjdh-2026-000074

10. Sampling	How were participants selected? e.g. purposive, convenience, consecutive, snowball	Purposive sampling was employed (Data collection in Methods section pg 5).
11. Method of approach	How were participants approached? e.g. face-to-face, telephone, mail, email	Face-to-face (Data collection in Methods section pg 5-6).
12. Sample size	How many participants were in the study?	In Results section pg 7.
13. Non-participation	How many people refused to participate or dropped out? Reasons?	In Results section pg 7.
Setting		
14. Setting of data collection	Where was the data collected? e.g. home, clinic, workplace	In health centres.
15. Presence of non-participants	Was anyone else present besides the participants and researchers?	No one was present besides the participants and researchers.
16. Description of sample	What are the important characteristics of the sample? e.g. demographic data, date	Characteristics of the sample were described (in Results pg 7).
Data collection		
17. Interview guide	Were questions, prompts, guides provided by the authors? Was it pilot tested?	Interview guides were developed based on relevant literature. The guides were pre-tested to ensure clarity and accuracy (Data analysis in Methods section pg 6-7).
18. Repeat interviews	Were repeat interviews carried out? If yes, how many?	No repeat interviews were carried out.
19. Audio/visual recording	Did the research use audio or visual recording to collect the data?	All interviews were recorded using a summary template (Data analysis in Methods section pg 5).

20. Field notes	Were field notes made during and/or after the interview or focus group?	Field notes were written in every interview to capture insights (Data analysis in Methods section pg 7).
21. Duration	What was the duration of the inter views or focus group?	The duration of interviews ranged from 15 to 30 minutes (Data collection in Methods section pg 6).
22. Data saturation	Was data saturation discussed?	Data saturation was not reported.
23. Transcripts returned	Were transcripts returned to participants for comment and/or correction?	Transcripts were not returned to participants.
**Domain 3: analysis and findings**		
Data analysis		
24. Number of data coders	How many data coders coded the data?	Two independent coders (Data analysis in Methods section pg 6-7).
25. Description of the coding tree	Did authors provide a description of the coding tree?	The coding tree was developed through NVivo 15® but not presented in the manuscript.
26. Derivation of themes	Were themes identified in advance or derived from the data?	Themes were derived from the data (Data analysis in Methods section pg 6-7).
27. Software	What software, if applicable, was used to manage the data?	NVivo 15® used (Data analysis in Methods section pg 7).
28. Participant checking	Did participants provide feedback on the findings?	Participants did not provide feedback on the findings.
Reporting		
29. Quotations presented	Were participant quotations presented to illustrate the themes/findings? Was each quotation identified? e.g. participant number	Quotations were presented and participant number provided (Table 2 pg 10-11).
30. Data and findings consistent	Was there consistency between the data presented and the findings?	Consistency was checked (in Results section).
31. Clarity of major themes	Were major themes clearly presented in the findings?	Major themes were clearly presented (Table 2 pg 10-11).
32. Clarity of minor themes	Is there a description of diverse cases or discussion of minor themes?	Minors themes were described (in Results section pg 10-11).
